# Effects of Sulforaphene on the Cariogenic Properties of Streptococcus Mutans In Vitro and Dental Caries Development In Vivo

**DOI:** 10.3390/antibiotics12091359

**Published:** 2023-08-24

**Authors:** Yuehong Zhou, Binhan Zhang, Yufei Wang, Rongdang Hu

**Affiliations:** 1 The College of Renji, Wenzhou Medical University, Wenzhou 325000, China; 2 State Key Laboratory of Oral Diseases, National Clinical Research Center for Oral Diseases, Department of Cariology and Endodontics, West China Hospital of Stomatology, Sichuan University, Chengdu 610041, China; 3 The College of Life Science, Sichuan University, Chengdu 610041, China

**Keywords:** *Streptococcus mutans*, Sulforaphene, isothiocyanates, biofilms, rat

## Abstract

Sulforaphene (SFE) is a common nutritional supplement with antibacterial, anti-cancer, and anti-inflammatory effects. However, the effects of SFE on the cariogenicity of *Streptococcus mutans* and dental caries have not been reported. The objectives of this study were to investigate the caries-controlling potential of SFE. The effects of SFE on *S. mutans* were investigated using the broth microdilution method, crystal violet staining, SEM observation, acid tolerance assays, lactic acid quantification, and polysaccharide measurements. A rat caries model was established to evaluate the caries-controlling effects and biocompatibility of SFE in vivo. SFE inhibited *S. mutans* growth and biofilm formation. Furthermore, SFE restrained the cariogenic properties of *S. mutans*, including its acid production, acid tolerance, and extracellular polysaccharide production, without affecting the bacterial viability at sub-inhibitory levels. In the rat caries model, SFE significantly arrested the onset and development of dental caries. Moreover, no visible hemolytic phenomenon or cytotoxicity was detected in the SFE groups. After four weeks of SFE treatment, all rats remained in apparent good health with no significant differences in weight gain; their hemogram and biochemical parameters were normal; no pathological changes were observed in the oral mucosa, liver, or kidneys. In conclusion, SFE was safe and inhibited the development of caries effectively.

## 1. Introduction

Dental caries, often known as tooth decay or cavities, represents one of the most prevalent diseases affecting individuals worldwide of all ages [1]. It is a chronic condition that gradually worsens over time, mainly due to bacterial activity, which causes the demineralization of the tooth enamel. *Streptococcus mutans* is a critical bacterium involved in the development of dental caries [2]. The principal steps in the induction of caries by *S. mutans* include bacterial attachment, plaque formation, acid production, enamel demineralization, and biofilm formation. Once attached to the tooth surface, *S. mutans* converts sugars and carbohydrates from the diet into lactic acid through glycolysis. These acids, produced by *S. mutans*, lower the pH level of the oral cavity significantly and lead to the demineralization of the tooth enamel [3]. Over time, *S. mutans* will transform into a biofilm, a complicated microbial community that adheres to tooth surfaces. This biofilm can protect *S. mutans* from antimicrobial treatments and the host’s immune responses, leading to chronic infection and continued tooth decay [4].

Effective dental caries management requires targeting these processes to disrupt the growth and survival of *S. mutans* and other cariogenic bacteria [5]. Research on the potential antibacterial properties and caries prevention effects of natural plant extracts has increased recently. Several plant extracts have been found to exhibit bacteriostatic or bactericidal effects against *S. mutans*, including green tea extract, aloe vera extract, cranberry extract, propolis extract, and others [6,7]. However, the safety and toxicity of these extracts at higher concentrations or with long-term use should also be carefully evaluated.

Cruciferous vegetables, such as radishes, cauliflowers, and broccoli, are important crops worldwide and are rich in isothiocyanates (ITCs; R-N=C=S) [8]. The three types of major ITCs found in cruciferous vegetables are aliphatic ITCs, aromatic ITCs, and Indolyl ITCs. Previous studies have primarily concentrated on how the antibacterial properties of ITCs can aid in the management of plant diseases and the preservation of food [9]. Sulforaphene (SFE, CH_3_-SO-CH=CH-(CH_2_)_2_-N=C=S) belongs to the aliphatic ITCs and possesses numerous pharmacological effects, including inhibiting cancer progression [10,11], ameliorating inflammation [12,13] and attenuating adipogenesis [14,15]. Furthermore, SFE is a low-toxicity substance commonly consumed with various dietary nutritional supplements. Its administration to humans typically exhibits good tolerability [16]. The antimicrobial properties of SFE have also been investigated, and SFE has been shown to inhibit *Helicobacter pylori* [17], *Cutibacterium acnes* [18], and several oral pathogens, including *S. mutans*, *Lactobacillus casei*, and *Candida albicans* [19]. However, the effects of SFE on the cariogenicity of *S. mutans* and dental caries have not been reported. In addition, any potential side effects or toxicity concerns regarding the use of SFE for caries prevention remain unknown.

Considering all of the above, we speculated that SFE could control the formation and progression of carious lesions by inhibiting the *S. mutans* growth and the *S. mutans* biofilms’ formation, as well as by restraining the cariogenic virulence factors of *S. mutans*. The objectives of this study were (1) to investigate the antimicrobial effects of SFE on planktonic *S. mutans* and *S. mutans* biofilms in vitro; (2) to investigate the impact of SFE on cariogenic virulence factors of *S. mutans* in vitro, specifically acid tolerance, acid production, and EPS generation; (3) to explore the caries-controlling effects of SFE in vivo, and (4) to verify the in vivo and in vitro biocompatibility of SFE. This study is the first to investigate the roles of SFE on the cariogenic properties of *S. mutans* in vitro and carious lesion progression in vivo.

## 2. Results

### 2.1. Evaluation of SFE’s Antibacterial Effects against S. mutans and Its In Vitro Cytotoxicity

**Figure 1:**
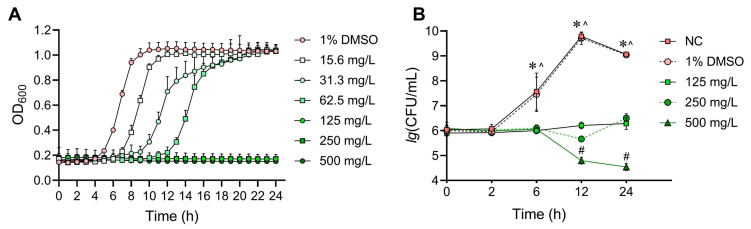
Effects of SFE on the growth of *S. mutans* UA159. (**A**) Kinetic growth curves of *S. mutans* in the presence of SFE. The OD_600_ values were recorded hourly using a microplate spectrophotometer. Values indicate means with standard deviations from three experiments. (**B**) Time-kill curves for SFE against *S. mutans* UA159. The living bacterial concentration values are expressed as *lg* (CFU/mL). Values are presented as means with standard deviations from three experiments. The symbols above the dots indicate significant differences within groups compared with the living bacterial concentration value at 0 h. *: non-treated control (NC) group; ^: 1% DMSO group; #: 500 mg/L SFE group.

SFE showed antibacterial effects against different *S. mutans* strains (Table 1). For six of the eight included *S. mutans* strains, the MIC value of SFE was 125 mg/L. However, we did not detect the MBC value of SFE in the tested concentration range. The effects of SFE on the growth of *S. mutans* UA159 were further evaluated by growth curves. As shown in Figure 1A, when exposed to sub-MIC levels of SFE (15.6–62.5 mg/L), *S. mutans* UA159 exhibited a prolonged lag phase and lower absorbance during the logarithmic phase. However, after 24 h, the mean absorbance of the sub-MIC groups did not significantly differ from that of the solvent control. It is not surprising that the OD_600_ values remained consistent with the initial level when 1- to 4-fold MICs of SFE were introduced to the bacterial cultures. This indicates that bacterial growth was completely inhibited. In order to clarify whether SFE has a bactericidal effect, time-kill kinetic curves were constructed. Figure 1B demonstrates that SFE at 1-fold or 2-fold MICs could only halt the proliferation of *S. mutans* and does not lower the number of viable bacteria. However, after 24 h of treatment with SFE at 500 mg/L, the number of bacteria decreased by roughly two orders of magnitude (99% of the viable *S. mutans* cells). These findings demonstrate the strong bacteriostatic activity of SFE, while *S. mutans* growth can only be postponed at sub-inhibitory concentrations of SFE.

**Figure 2:**
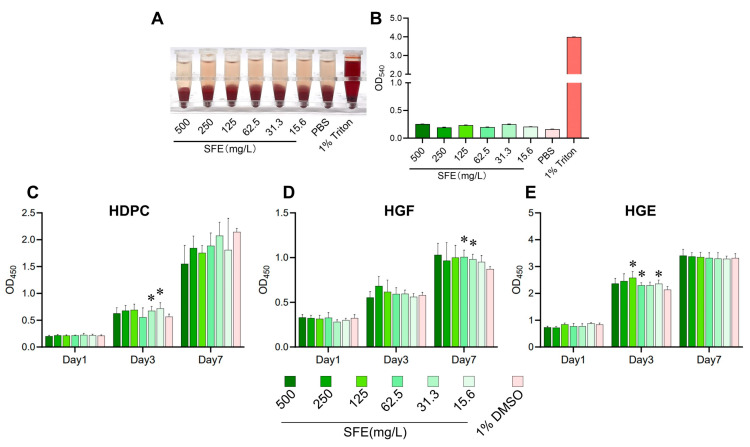
In vitro cytotoxicity evaluation of SFE. For hemolysis assays, the red cells were incubated at 37 °C for 30 min. (**A**) Representative hemolysis images of PBS (negative control), 1% Triton X-100 (positive control), and SFE at varied concentrations; (**B**) Hemolytic toxicity of SFE was tested by measuring OD_540_ values. The experiment was repeated three times. (**C**–**E**) The effects of SFE on cell proliferation were evaluated using Cell Counting Kit-8 (CCK-8) assays. The OD_450_ value is used to reflect cell proliferation. (**C**) Effects of SFE on the cell proliferation of human dental pulp cells (HDPCs); (**D**) Effects of SFE on the cell proliferation of human gingival fibroblasts (HGFs); (**E**) Effects of SFE on the cell proliferation of human gingival epithelial cells (HGEs). Values indicate means with standard deviations from three experiments. The asterisk represents *p* < 0.05.

To determine the in vitro cytotoxicity of SFE, a hemolysis assay on mammalian erythrocytes and cytotoxicity assays were conducted using three common oral cells, which included human dental pulp cells (HDPCs), human gingival fibroblasts (HGFs) and human gingival epithelial cells (HGEs). Figure 2A,B demonstrated that there was no visible hemolytic phenomenon, and erythrocytes were not significantly ruptured by SFE in concentrations of up to 500 mg/L. The cytotoxicity of SFE on HDPCs, HGFs, and HGEs was evaluated by Cell Counting Kit-8 (CCK-8) assays. The results are illustrated in Figure 2C–E. No cytotoxicity was observed on day 1 for any concentration on any cells. In addition, a significant increase in the OD_450_ value was observed in the SFE groups on day 3 and day 7, indicating that SFE enhanced cell proliferation to varying degrees. The hemolysis assay and cytotoxicity assay results demonstrated the favorable security of SFE.

### 2.2. Evaluation of SFE’s Effects on Cariogenic Virulence Factors and Biofilm Formation of S. mutans

**Figure 3:**
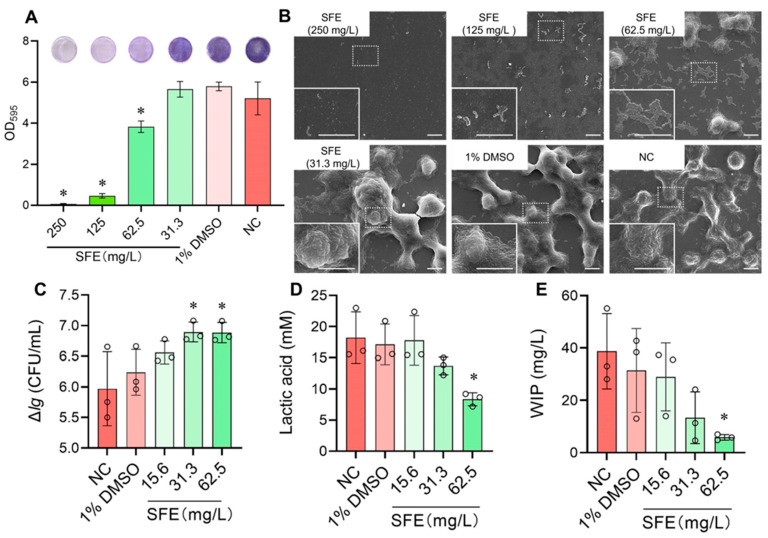
Evaluation of SFE’s effects on the biofilm formation and cariogenic virulence factors of *S. mutans*. (**A**) *S. mutans* biofilm formation after 24 h was quantified using crystal violet dye. The values indicate means with standard deviations from three experiments, and the typical pictures of stained biofilms in one of the three repeated assays are presented above the bars; (**B**) *S. mutans* biofilms morphology treated with SFE for 24 h (magnification: 3000×). High magnification images (20,000×) of the white rectangular area are shown in the lower left corner. The bar represents 10 μm; (**C**) Effects of SFE on acid tolerance of *S. mutans*. The value of Δ*lg* (CFU/mL) reflects the difference of *S. mutans* CFU counts before and after the 2 h low pH culturing; (**D**) Lactic acid production of *S. mutans* treated with varied concentrations of SFE for 24 h; (**E**) The amounts of water-insoluble polysaccharides (WIPs) of *S. mutans* biofilms treated with various concentrations of SFE for 24 h. Values (○) indicate means with standard deviations from three experiments. The asterisk represents (*) *p* < 0.05. NC: non-treated control.

As shown in Figure 3A, SFE had dose-dependent inhibitory effects on biofilm formation. The *S. mutans* biofilm development was first significantly suppressed by SFE at a concentration of 62.5 mg/L (*p* < 0.05). The SEM observations (Figure 3B) showed that the extracellular matrix somewhat decreased after treatment with 62.5 mg/L of SFE. When the SFE levels were higher than 125 mg/L, no molded biofilm was detected. The bacteria appeared to be planktonic and were dispersed throughout. The Δ*lg* (CFU/mL) values illustrate the change in the living *S. mutans* cell counts after two hours of cultivation in a lethal acidic environment (pH of 5.0) and thus reflect the changes in the acid tolerance of *S. mutans*. The increase in the Δ*lg* (CFU/mL) of SFE at 31.3 mg/L (quarter-MIC) and 62.5 mg/L (half-MIC) at pH 5.0 was significant (*p* < 0.05), according to Figure 3C. We determined the effects of SFE on the acidogenicity of *S. mutans* by analyzing the changes in *S. mutans*’s lactic acid production. Figure 3D demonstrates that the lactic acid generation in the *S. mutans* culture was significantly suppressed (*p* < 0.05) when SFE was applied at a concentration of 62.5 mg/L. The levels of water-insoluble polysaccharides (WIPs) were significantly decreased by the half-MIC of SFE (*p* < 0.05), as shown in Figure 3E. These results showed that SFE decreased the aciduricity, the acidogenicity, and the capacity to produce WIPs of *S. mutans* at sub-MIC concentrations.

**Figure 4:**
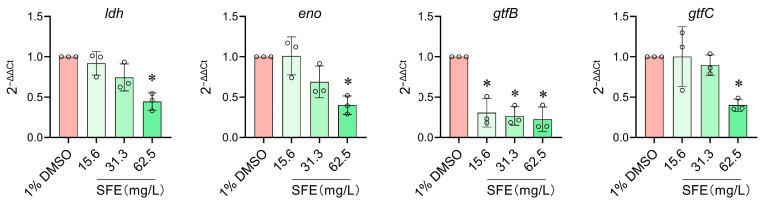
Evaluation of SFE’s effects on virulence gene expression in *S. mutans.* The gene *ldh* encodes lactate dehydrogenase, the gene *eno* encodes enolase, and the genes *gtf*BC encode glucosyltransferase. The reference gene: 16S rRNA. Values (○) indicate means with standard deviations from three experiments. The asterisk represents (*) *p* < 0.05.

The expression of cariogenic virulence genes (*ldh*, *eno*, *gtf*B, and *gtf*C) in *S. mutans* treated with SFE at sub-MIC levels was determined by reverse transcription and quantitative real-time PCR (RT-qPCR). As shown in Figure 4, the half-MIC of SFE (62.5 mg/L) significantly downregulated the expression of the acid production-related genes *ldh* and *eno* (*p* < 0.05), which was consistent with the results of the lactic acid production assay (Figure 3D). Moreover, SFE at 62.5 mg/L significantly suppressed the expression of *gtf*B and *gtf*C, which encode glucosyltransferase, an enzyme critical for WIP generation. Thus, SFE inhibited the biofilm formation and cariogenic factors of *S. mutans* at both the phenotypic and transcriptional levels.

### 2.3. Evaluation of SFE’s Effects on Carious Lesion Formation and Progression In Vivo

**Figure 5:**
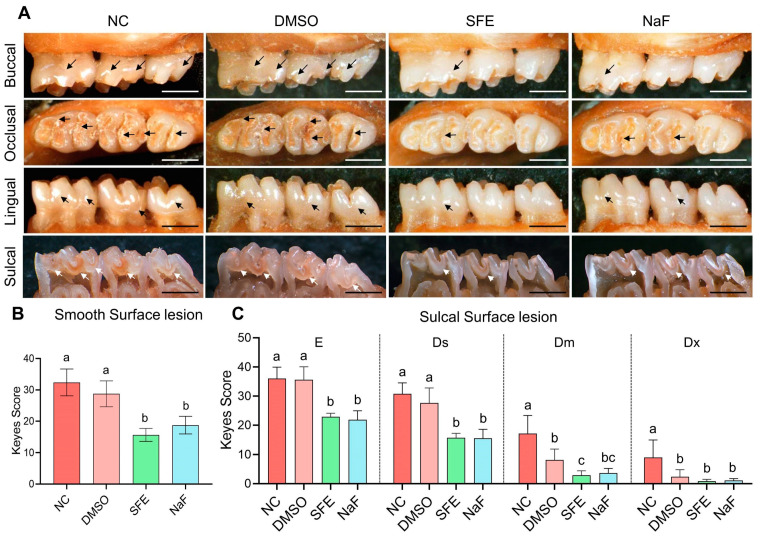
In vivo effects of SFE against the development of carious lesions. A rat caries model was established. The topical treatment was applied to the molars of the rats using small dental brushes dipped in each group of solutions. Consequently, the rats’ teeth were brushed for 5 min three times daily for 4 consecutive weeks. Each rat received 100 μL of solution during each treatment session. (**A**) Typical images of teeth of rats from different groups. Typical red carious lesions are denoted by arrows. Bar = 500 μm; (**B**) Smooth surface caries assessment by Keyes scoring method. (**C**) Sulcal surface caries assessment by Keyes scoring method. Sulcal surface caries were divided into four degrees: enamel only (E), less than 1/4 of the dentin affected (slightly dentinal, Ds), 1/4~3/4 of the dentin affected (moderate dentinal, Dm), beyond 3/4 of the dentin affected (extensive dentinal, Dx). Values indicate means with standard deviations from eight rats from each group. A significant difference between groups is denoted by different letters (*p* < 0.05). NC means non-treated control. DMSO means solvent control (1% DMSO). SFE means applying 125 mg/L of SFE. NaF means applying 500 ppm of F^−^.

A validated rat caries model was used to investigate the hypothesis of whether topically delivering SFE can avert the onset and progression of dental caries in vivo. In this model, as indicated by the arrows in Figure 5A, carious lesions (colored red), gradually formed on teeth. Extensive smooth-surface and sulcal-surface lesions were substantially more prevalent in the non-treated control (NC) and 1% DMSO groups. As shown in Figure 5B, a topical treatment of SFE (125 mg/L) and fluoride (500 ppm of F^−^) decreased the incidence of smooth-surface caries. Out of all these groups, the SFE group received the lowest score for smooth-surface lesions. The topical application of SFE or fluoride also decreased the occurrence and severity of sulcal-surface caries, as demonstrated in Figure 5C. Notably, the Keyes scores of the SFE and fluoride groups did not vary statistically.

### 2.4. Evaluation of the In Vivo Biological Safety of SFE

**Figure 6:**
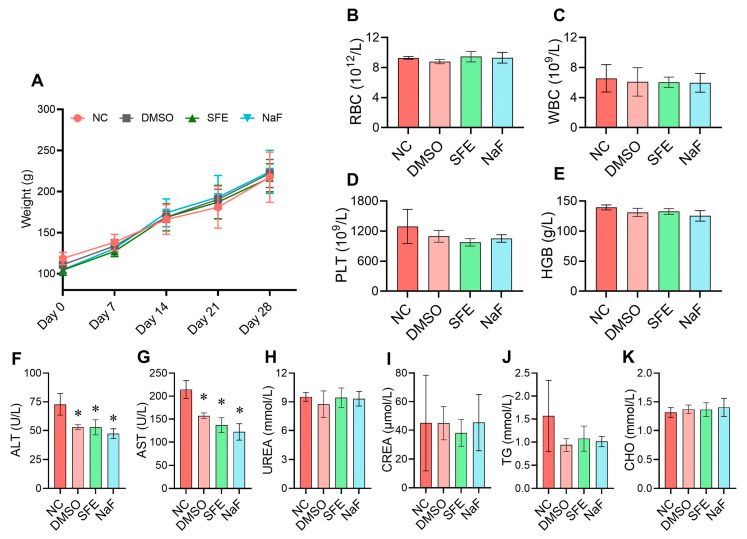
In vivo biological safety evaluation of SFE. The health condition of the rats in the caries model was evaluated. (**A**) Rats’ weight growth during the four-week treatment period; (**B**–**E**) The hemogram parameters at the treatment termination point (day 28) were listed as red blood cells (RBCs, (**B**)), white blood cells (WBCs, (**C**)), platelet count (PLT, (**D**)), and hemoglobin concentration (HGB, (**E**)); (**F**–**K**) The serum clinical biochemical parameters at the treatment termination point (day 28) were listed as alanine aminotransferase (ALT, (**F**)), aspartate aminotransferase (AST, (**G**)), blood urea (UREA, (**H**)), creatinine (CREA, (**I**)), triglycerides (TG, (**J**)) and cholesterol (CHO, (**K**)). The means and standard deviations of the data from 8 rats are displayed. The asterisk denotes a *p*-value less than 0.05. NC means non-treated control. DMSO means solvent control (1% DMSO). SFE means applying 125 mg/L of SFE. NaF means applying 500 ppm of F^−^.

In vivo biocompatibility testing of SFE was also conducted using the rat model. During the 4-week examination period, all rats were observed to be in good health. There were no unexpected fatalities, and as shown in Figure 6A, no significant differences in weight gain were noted among any of the treatment groups (*p* > 0.05). Figure 6B–E showed that there were no noticeable distinctions between the control group and the SFE-treated groups in terms of red blood cells (RBCs), white blood cells (WBCs), platelet counts (PLTs), or hemoglobin values (HGBs) (*p* > 0.05). The levels of the enzymes alanine aminotransferase (ALT) and aspartate aminotransferase (AST) in the blood serum did not significantly change after treatment with SFE, NaF, or DMSO in comparison to the NC group (*p* > 0.05), suggesting no liver damage or liver infections (Figure 6F,G). The use of SFE did not result in substantial modifications in the serum levels of blood urea (UREA), creatinine (CREA), triglyceride (TG), and cholesterol (CHO) compared to the control group after the 4-week experiment (Figure 6H–K).

**Figure 7:**
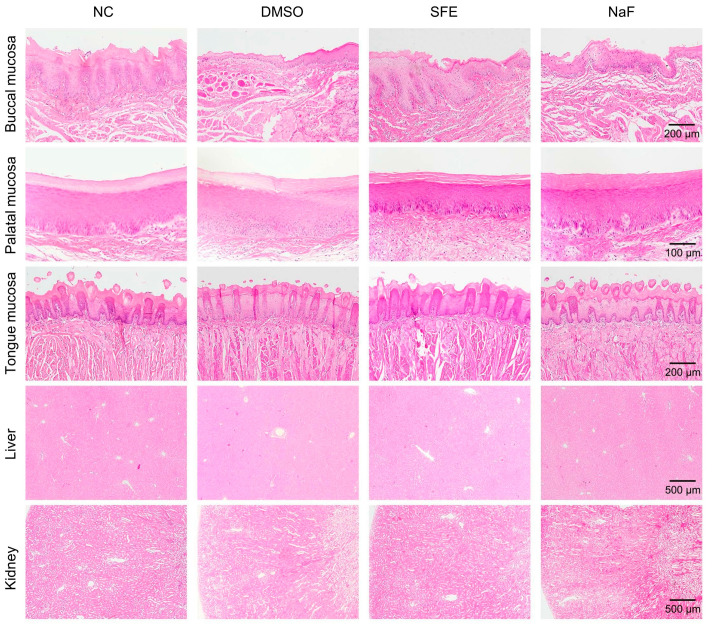
Histopathology of the oral mucosa, liver, and kidney tissues in rats after a four-week treatment. NC means non-treated control. DMSO means solvent control (1% DMSO). SFE means applying 125 mg/L of SFE. NaF means applying 500 ppm of F^−^.

Additionally, after a 4-week topical treatment, Figure 7 illustrates that the epithelial keratinized layers of the oral mucosa tissue treated with SFE or other groups were intact and clear, with no indications of cytopathic changes, deformation, hyperemia, hydrops, erosion, or ulcer formation. Additionally, no discernible leukocyte infiltration was detected in the dermis or submucosa of all groups. No significant changes were observed in the collagen fibers, muscle fibers, or blood vessels of all groups. Furthermore, in the slices of liver and kidney tissues, the liver lobules and glomeruli were clear and intact in all groups. The structural patterns of the treated groups’ kidneys and liver showed no apparent organ abnormalities, inflammatory infiltration, or lesions compared to those of the NC group. In summary, according to the above comprehensive evaluation, SFE did not show adverse side effects or systemic toxicity on the rat’s health.

## 3. Discussion

In-depth research has been conducted on the utilization of materials made from plants as antibacterial agents. The most prevalent secondary metabolites in the botanical order Brassicales are glucosinolates, which are converted into ITCs through enzymatic hydrolysis [20]. Apart from SFE, the reported aliphatic ITCs included sulforaphane, iberin, erucin, allyl ITCs, and hexyl ITCs. Ko et al. [19] reported that SFE and sulforaphane both showed the lowest MIC among all the tested ITCs against *S. mutans*. Notably, SFE showed higher antimicrobial activity against *C. albicans* than sulforaphane, and the authors deduced that the double bond in SFE’s chemical structure might account for this activity [19]. Because of this structural feature, we selected SFE from a wide range of ITCs for further study.

The antimicrobial properties of SFE on planktonic *S. mutans* and *S. mutans* biofilms in vitro were examined first in the current work. Then the caries-controlling effects of SFE in vivo were explored, and the in vivo and in vitro biocompatibility of SFE was verified. The results demonstrated that SFE is safe and may hinder *S. mutans* from growing and forming biofilms. In addition, at the sub-MIC levels, SFE can weaken the cariogenic virulence factors of *S. mutans* at both the phenotypic and transcriptional levels. The above effects make it possible for SFE to reduce the onset and progress of dental caries in vivo. To our knowledge, this study represents the first investigation into the efficacy of SFE in controlling caries. Furthermore, it scrutinizes the oral biocompatibility of SFE, both in vitro and in vivo.

Using the broth microdilution method, we determined that the MIC value of SFE was 125 mg/L (0.713 mM, Table 1). Additional time-kill kinetic curves (Figure 1B) showed that a high concentration of SFE killed 99% of the viable *S. mutans* cells. From an objective perspective, SFE’s MIC values are higher than those of CHX (Table 1), classical antibiotics [21,22], antimicrobial peptides [23,24], and quaternary antimicrobials [25], whose MIC values are often at μM levels. SFE, on the other hand, as a natural component, has the advantage of being safer, more widely sourced, and more easily accessible. The antibacterial effects of SFE are comparable to those of tea polyphenols, among numerous natural antimicrobial compounds [26,27]. Moreover, SFE has the most superior antibacterial properties among ITCs [19].

Caries management agents should successfully prevent the creation of new biofilms because cariogenic bacteria often grow in biofilms, which helps them resist antimicrobial agents [28,29]. *S. mutans* biofilm formation was significantly restrained by SFE (Figure 3A,B). SFE’s MBIC_50_ value in the current study was equal to half of its MIC, while its MBIC_90_ value was the same as its MIC (Figure 3A). We speculated that the inhibitory effect of SFE above MIC levels on biofilm formation might primarily be through the prevention of bacterial growth. Further investigation revealed that using SFE at 1/2 MIC not only delays the growth of bacteria (Figure 1A), which slows down the formation of biofilms but also directly decreases the number of biofilms by preventing WIP production (Figure 3E).

The production of plaque biofilms is facilitated by the EPS, particularly WIPs. And the WIPs produced by *S. mutans* also facilitated the attachment of other oral bacterial [28]. In our experiments, we discovered that SFE significantly and dose-dependently reduced the formation of WIPs at sub-MIC levels. Extracellular glucosyltransferases (GTFs) are key enzymes in EPS synthesis. Highly insoluble glucans that comprise the scaffold of the EPS matrix are produced by GTFB (encoded by *gtf*B), and GTFC (encoded by *gtf*C) produces both soluble and insoluble glucans, which shape the early EPS layers and provide binding sites for *S. mutans* [30]. Using RT-qPCR (Figure 4), it was shown that SFE could suppress the expression of *gtf*BC, which might be the primary mechanism by which SFE prevents WIP synthesis.

*S. mutans* is more prevalent in the cariogenic plaque because it exhibits acidogenic and aciduric characteristics, making it a stronger competitor than species that are less acid-tolerant in challenging environmental conditions [31]. Sub-MIC doses of SFE inhibited *S. mutans* from producing acid in vitro, and this suppression was dose-dependent (Figure 3D). The primary pathway by which acid is produced in *S. mutans* is glycolysis. Enolase, which is encoded by the gene *eno*, catalyzes the conversion of 2-phosphoglycerate into phosphoenolpyruvate, a crucial rate-limiting step in the transmembrane transport of carbohydrates [32]. Lactate dehydrogenase (LDH) catalyzes the conversion of pyruvate to lactate, which is the last stage in glycolysis [33]; hence, *ldh* expression and lactic acid production are closely related processes. At half-MIC levels, SFE can downregulate the expression of *eno* and *ldh* (Figure 4), thereby impeding the transport of sugar substrates and lactic acid production. This might explain the way SFE suppresses acid production in *S. mutans*.

As for the aciduricity, SFE showed a strong ability to reduce the acid tolerance of *S. mutans* at pH 5.0 when used at 1/4 and 1/2 of its MIC (Figure 3C). The higher stability of SFE in a low pH environment might account for this. As Song et al. [34] proved, the degradation reaction that leads to SFE breakdown was effectively inhibited at a low pH. In addition, the weakened aciduricity might also be related to the decreased activity of LDH. NADH is converted to NAD+ by LDH when it catalyzes the conversion of pyruvate to lactate in anaerobic conditions. Therefore, inhibiting LDH would also lead to the accumulation of NADH, which would have toxic effects on *S. mutans* [35].

The Keyes’ scoring system and the rat caries model offer the most thorough and detailed information for caries incidence, including the position and depth of the lesions [36]. In comparison to the NC group, the application of 125 mg/L of SFE for 5 min three times a day resulted in significantly lower scores for both smooth-surface and sulcal-surface lesions (Figure 5). Due to the capacity to alter bacterial bioactivity and restore the equilibrium between demineralization and remineralization, fluoride is still regarded as the cornerstone of caries healthcare [37]. It is worth noting that there was no statistically significant difference between the Keyes scores of the SFE group and those of the NaF group (500 ppm of F^−^). The above findings showed the promising anticaries efficacy of SFE.

Any potential side effects or toxicity concerns will obstruct the application of SFE for caries management. Our study demonstrated the satisfactory biocompatibility of SFE. In the current work, the results indicated that SFE had no noticeable impact on erythrocyte rupture under the concentrations used (less than 500 mg/L). Additionally, there was no evidence of any harmful effects on three commonly found oral cells—HDPCs, HGFs, and HGEs—regardless of the concentration used (Figure 2). Before our studies, Byun et al. [38] discovered that SFE showed no toxicity toward nonmalignant cells while inhibiting the proliferation of the human colon cancer cell lines, which is consistent with our findings. Furthermore, the SFE did not have any adverse side effects or systemic toxicity on the health of rats. Hemogram parameters reflect adverse conditions such as anemia, inflammation, and other blood system disorders [39]. The liver function was evaluated using ALT and AST, and the kidney function was reflected by UREA and CREA [40]. TG and CHO were also measured to assess whether the high-sugar diet (cariogenic diet) affected the blood lipids in the rats [41]. During the 4-week experiment, the use of SFE did not result in substantial modifications to the ALT, AST, UREA, CREA, TG, or CHO values. The structural patterns of the treated groups’ kidneys and liver showed no apparent organ abnormalities or lesions. These results demonstrated that SFE did not have any adverse side effects or systemic toxicity on the health of rats.

In conclusion, SFE dramatically delayed the occurrence and progression of dental caries in the rat caries model by exhibiting outstanding multiple-effects activity against *S. mutans* and its cariogenic properties. It did not result in side effects or systemic toxicity on the health of rats. All results suggest that SFE is safe and possesses multiple effects for caries control. It would be worthwhile to conduct more research to promote the clinical application of SFE, such as research on the preformed biofilms, antimicrobial mechanisms, better application approaches, and treatment regimens for SFE in the oral cavity, as well as the synergistic effects with other caries-controlling agents.

## 4. Materials and Methods

### 4.1. Bacterial Strains, Cells, and Growth Conditions

*S. mutans* UA159 was purchased from the American Type Culture Collection (Manassas, VA, USA). Another strain type, *S. mutans* GS-5, was bought from the Guangdong Culture Collection Center. The clinically isolated *S. mutans* strains (COCC31-8, COCC32-3, COCC33-4, COCC33-17, COCC33-14, and COCC33-8) were isolated and identified previously from the West China Hospital of Stomatology (Chengdu, China). All *S. mutans* strains were cultured under anaerobic conditions (85% N_2_, 10% H_2_, and 5% CO_2_) at 37 °C using brain-heart infusion broth (BHI; Oxoid, Basingstoke, Hampshire, UK) [24]. All the strains were incubated overnight till the logarithmic phase before use. Under our experimental conditions, the bacterial concentration was calculated by multiplying the OD_600_ value by 3.12 × 10^9^ CFU/mL. As previously described [42], buffered peptone water (BPW, Nissui, Tokyo, Japan) was used for bacterial culture in the lactic acid production measurement. Modified mitis salivarius–bacitracin agar (MSB, HB8912-1, Hopebio, Qingdao, China) was used for the selective culture of *S. mutans* [43]. The human dental pulp cells (HDPCs), human gingival fibroblasts (HGFs), and human gingival epithelial cells (HGEs) used in the cytotoxicity test were purchased from the Procell Life Science and Technology (Wuhan, China). The cells were cultured at 37 °C with 5% CO_2_ using Dulbecco’s modified Eagle’s medium (DMEM; Gibco^TM^, Invitrogen, Carlsbad, CA, USA) containing 10% fetal bovine serum (Gibco^TM^) and a 1% penicillin–streptomycin solution (Gibco^TM^).

### 4.2. Bacterial Susceptibility Assay

A modified broth microdilution method was used to assess the MIC and MBC of SFE in 96-well microtiter plates [24]. SFE (20.0 μL in each well) was prepared in 10% (*v*/*v*) DMSO at concentrations ranging from 5000 to 156.25 mg/L as two-fold serial dilutions. Then, 180.0 μL of a bacterial culture in BHI broth was added to each well. The final concentration of the bacterial culture was 1.0 × 10^7^ CFU/mL. The final SFE concentrations in 1% DMSO, ranging from 500 to 15.625 mg/L, were present in each microwell. The solvent control was 1% DMSO, the blank control was BHI broth, and the positive control was chlorhexidine (CHX). After an anaerobic incubation at 37 °C for 48 h, the OD_600_ values of all microwells were measured by the microplate spectrophotometer (Multiskan GO, Thermo Scientific, Waltham, MA, USA). The lowest SFE concentration that had identical OD_600_ values to the blank BHI broth well was considered to be the SFE’s MIC. Then, aliquots (100.0 μL) from the wells that had no visible bacterial growth were pipetted and spread onto BHI agar for the MBC assays. Anaerobic incubation of these agar plates took place at 37 °C for 48 h. The MBC of SFE was determined to be the lowest concentration that totally stopped bacterial growth on the BHI agar. These assays were repeated six times.

### 4.3. Kinetic Growth Curve Assays

The 24-h growth curves were measured using a BioTek LogPhase 600 microbiology reader (Agilent, Santa Clara, USA). As described in Section 4.2, in each well of 96-well microtiter plates, two-fold serial dilutions of SFE (20.0 μL in each well) were mixed with 180.0 μL of an *S. mutans* UA159 culture. The range of the final SFE concentrations was from 500 to 15.625 mg/L. The final concentration of the bacterial culture was 1.0 × 10^7^ CFU/mL. The solvent control was 1% DMSO. Light mineral oil (M5904, Sigma) was added at 50.0 μL per well as a vapor barrier. The plates were shaken at 500 rpm while being incubated anaerobically for 24 h at 37 °C. The optical density at 600 nm (OD_600_) was recorded every hour. The experiments were independently repeated three times.

The bactericidal kinetics of SFE against the *S. mutans* UA159 were assessed using a time-kill assay, as described previously [27]. Briefly, SFE was added to the bacterial cultures at final concentrations equal to 1-fold MIC (125 mg/L), 2-fold MIC (250 mg/L), and 4-fold MIC (500 mg/L). Subsequently, anaerobic incubation was carried out at 37 °C. The solvent control was 1% DMSO. At 0, 2, 6, 12, and 24 h, 100 μL aliquots of suspension were withdrawn and serially diluted 10-fold with PBS. For the CFU counting, 100 μL of each dilution was plated on BHI agar and incubated anaerobically at 37 °C for 48 h. Time-kill kinetic curves were constructed by plotting *lg* (CFU/mL) versus incubation time over 24 h. The assays were performed in triplicate on three different days.

### 4.4. In Vitro Biocompatibility Assay

The cell experiments were approved (WCHSIRB-D-2022-366). The cytotoxicity test was conducted as previously described [44]. HDPC (CP-H240, Procell), HGF (CP-H231, Procell), and HGE (CP-H203, Procell) were seeded in 96-well plates at a concentration of 1 × 10^3^ cells per well. After the first 48 h-culture, the plates were exposed to medium (100 μL) containing SFE at concentrations of 15.6 to 500 mg/L for 1, 3, or 7 days. PBS was used as the negative control. The solvent control was 1% DMSO. At the set time points of the exposure period, a Cell Counting Kit-8 (CCK-8; Beyotime, Shanghai, China) was used to assess the proliferation of the SFE-treated cells. Media (100 μL) containing 10% (*v*/*v*) CCK-8 was added to the wells, and the plates were incubated for 1 h away from light to allow the formation of formazan. The OD_450_ was detected using a microplate-spectrophotometer to reflect cell proliferation. These experiments were repeated three times.

The hemolytic activity of SFE was further investigated. The off-fiber sheep blood (1001339-1, Hopebio) was centrifuged at 2000× *g* for 5 min to collect the red cells [45]. The red cells were incubated in PBS (1 mL) containing SFE at 37 °C for 30 min. The negative control was PBS, and Triton X-100 (1%) served as the positive control. The OD_540_ values of the supernatants were measured using a spectrophotometer following centrifugation (2000× *g*, 10 min). The experiment was repeated three times.

### 4.5. Experiments of Biofilm Formation Inhibition

A crystal violet staining assay was conducted as previously described [24]. The SFE (100.0 μL in each well) was added to a 24-well plate with a gradient dilution*. S. mutans* UA159 cells in BHIS (BHI plus 1% sucrose) were then added into the wells (900.0 μL) with a final concentration of 1.0 × 10^6^ CFU/mL. The negative control was an untreated bacterial culture, and the solvent control was 1% DMSO. Three replicates were set to the control groups and experimental groups. The biofilm formed during the 24 h-incubation anaerobically. Then, the supernatant was aspirated, and the bottom biofilms were washed twice with PBS to remove planktonic bacteria. After washing, the biofilm was fixed with 4% paraformaldehyde for 15 min. Then, crystal violet (0.1%) was added to stain the biofilms for 5 min. The stained biofilms were photographed under a stereomicroscope (Leica, Wetzlar, Germany). For biofilm formation quantification, anhydrous ethanol (500 μL) was added to each well to redissolve the crystal violet dyes. The absorbance at 595 nm (A_595_) was measured using a microplate spectrophotometer. The experiment was repeated three times.

### 4.6. Biofilm Morphology Observations

Glass slides were placed into the 24-well plate to allow *S. mutans* biofilm formation. The biofilm culture method was the same as in Section 4.5. The biofilm was washed with PBS buffer, fixed with 4% paraformaldehyde for 2 h, and dehydrated with a gradient of ethanol (25%, 50%, 75%, 90%, and 100%, each for 30 min). The biofilm samples on glass slides were then critical point dried; gold was sputter coated onto them, and the biofilm morphology was examined using scanning electron microscopy (SEM; Apreo 2S, Thermo Scientific) [46]. The experiment was repeated three times, and the representative images were selected and shown.

### 4.7. Lethal Acid Tolerance Test

Changes in the living *S. mutans* UA159 cells after 2 h exposure to pH 5.0 were measured to evaluate the effect of SFE on the acid tolerance of *S. mutans* [42]. *S. mutans* UA159 had been cultured in the tryptone glucose yeast extract (TYEG) broth up until the mid-log phase. After that, *S. mutans* was resuspended in TYEG broth buffered with 40 mM phosphate citrate buffer (pH 5.0). The final concentration of *S. mutans* was adjusted to 1.0 × 10^8^ CFU/mL. The experimental groups used the medium containing different concentrations of SFE to resuspend the bacteria, while an untreated bacterial suspension was used as the negative control, and 1% DMSO was used as the solvent control. The 100 μL of samples were taken out for a 10-times dilution and CFU counting before and after the 2-h incubation at pH 5.0. The value of Δ*lg* (CFU/mL) reflects the difference of *S. mutans* CFU counts before and after the 2 h low pH culturing. Δ*lg* (CFU/mL) = *lg* [(CFU counting) _initial_ − (CFU counting)_2 h_]. The experiment was repeated three times.

### 4.8. Lactic Acid Measurement

The biofilm culture method was the same as in Section 4.5. After the 24 h-biofilm was formed, BPW supplemented with 0.2% sucrose (1.5 mL) was added into the wells of a 24-well plate to allow the biofilm to produce lactic acid [42]. This plate continued to be incubated at 37 °C for another 2 h. The lactic acid assay kit (A019, Jiancheng, Nanjing, China) was used to measure the lactic acid concentration in the supernatants. According to the product manual, the A_570_ values were recorded, and lactic acid concentrations were calculated using standard curves [42]. The experiments were repeated three times independently.

### 4.9. Water-Insoluble Polysaccharides (WIPs) Measurement

The biofilm culture method was the same as in Section 4.5. After the 24 h incubation, the biofilms were then collected in 1 mL PBS buffer. The biofilm suspensions were centrifuged and washed twice with 1 mL sterilized deionized water to remove the water-soluble polysaccharide. The biofilm sediment was further resuspended in 1 mL of 0.4 M NaOH to extract the WIPs. The WIP supernatant was mixed with the anthrone reagent (0.1 g anthrone dissolved in 100 mL of 98% sulfuric acid, prepared before use) in a ratio of 1:3 (*v*/*v*), and the mixture was immediately placed in a dry heater preheated to 95 °C and reacted accurately for 6 min. The A_625_ was recorded after the reaction solution had returned to room temperature. Using standard curves, the concentration of WIP was calculated [47]. The WIP measurements were repeated three times.

### 4.10. RNA Isolation, Reverse Transcription, and Quantitative Real-Time PCR (RT-qPCR)

The expression of the genes responsible for cariogenic virulence was discovered using RT-qPCR. *S. mutans* UA159 was cultured until the late exponential phase in BHI broth with the addition of sub-MIC levels of SFE. The solvent control was *S. mutans* cultured in BHI containing 1% DMSO. The RNApure Bacteria Kit (CW0539, CWbio, Suzhou, China) was used for RNA isolation and purification. Complementary DNAs (cDNAs) were synthesized using the PrimeScript™ RT reagent Kit with gDNA Eraser (RR047A; Takara, Shiga, Japan). Table 2 includes a list of the tested genes and specific primers. Each 25 μL of PCR reagent contained 10 μL of TB Green^®^ Premix Ex Taq ™ II (RR820A; Takara), 80 ng of cDNA, and forward and reverse gene-specific primers (10 μM, 0.8 μL each). The LightCycler480 system (Roche Diagnostics, Rotkreuz, Switzerland) was used for the qPCR reaction. A two-step PCR amplification standard procedure was employed, with thermocycling settings of 95 °C for 5 s → 60 °C for 20 s over 40 cycles. The Ct values were measured after ensuring the amplification curves were normal, and the melting curves were all single peaks. To calculate the gene expression fold change, the 2^−ΔΔCt^ method was used and different gene expressions were normalized to the levels of 16S rRNA gene transcripts. The independent experiments were conducted three times.

### 4.11. Establishment of the Rat Caries Model

The animal experiments were reviewed and approved by the Institutional Review Board of the West China Hospital of Stomatology (WCHSIRB-D-2022-520). The environment and facility conditions of the laboratory animals followed the GB14925-2010 and ARRIVE guidelines 2.0 [51]. Thirty-two male specific-pathogen-free (SPF) Sprague–Dawley (SD) 17-day-old rats were provided by the Animal Experimental Center of Sichuan University. To suppress endogenous flora infection and help S. mutans settlement, the rats were maintained on a normal diet supplemented with antibiotics (0.1% ampicillin, 0.1% chloramphenicol, 0.1% carbenicillin, and 4000 U/mL penicillin in water) for three days [43]. A logarithmic-phase (10^8^ CFU/mL) S. mutans solution (100 μL) was injected orally into the bilateral outer cheeks of the rats continuously for three days to induce S. mutans infection. At the same time, the animals were provided with a cariogenic diet 2000# (56% sucrose; Trophic, Nantong, China) and 5% sucrose water ad libitum. The infection was confirmed by MSB agar [43]. The date of confirming S. mutans settlement was set as day 0. On the next day (day 1), the rats infected with S. mutans were randomly divided into four groups of 8 rats each, according to different treatment: (1) untreated control group (NC); (2) positive control group: NaF solution (500 ppm of F^−^); (3) solvent control group: sterilized 1% DMSO; and (4) treatment group: SFE solution (125 mg/L). To brush the rats’ teeth, specialized small disposable dental brushes (Φ = 1 mm; Kangjie Medical, Putian, China) were utilized to dip the solution of different groups. This topical treatment was performed for five minutes, three times per day, over four consecutive weeks. Each rat received 100 μL of solution during each treatment session. The weight of each rat was measured weekly, and their physical states were recorded. On day 28, venous blood samples were collected through a subclavian vein puncture under anesthesia [52]. Two blood samples were collected from each rat, one was immediately mixed with EDTA-K_2_ for hemogram parameters tests, and the other was left at room temperature for 2 h to extract the serum. The animals were euthanized by CO_2_ asphyxiation after the blood sampling.

### 4.12. In Vivo Biological Safety Evaluation

Within 2 h of collecting the venous blood samples, the hemogram parameters were examined using an automated instrument (TEK-VET5, Tecom, Nanchang, China). Centrifugation (1000× g, 15 min, 4 °C) was used to remove blood clots and separate the serum. The serum was tested for clinical biochemical parameters, which included alanine aminotransferase (ALT), aspartate aminotransferase (AST), blood urea (UREA), creatinine (CREA), triglyceride (TG), and cholesterol (CHO). All clinical biochemical analyses were performed using a biochemical blood analyzer (BK-200, BIOBASE, Ji’nan, China).

The liver, kidneys, and oral mucosal tissues (cheek, palate, and tongue) were aseptically dissected for histopathological assessment (hematoxylin-eosin staining) [53]. All samples were fixed for 24 h in 4% paraformaldehyde (Solarbio) and cut into sections. The slides were subjected to H& E staining. A digital slide scanning system (PRECICE, Beijing, China) was used to scan the slides. Subsequently, the image analysis was performed using CaseViewer 2.1 (3DHISTECH, Budapest, Hungary).

### 4.13. Keyes Score Assessment

The maxillary and mandibular bones were removed aseptically, and the soft tissue attached to the surface was completely removed. After fixation in 4% paraformaldehyde, all the jaws were cleaned, dried, and stained with a 0.4% ammonium oxalate solution for 12 h. According to the Keyes‘ criteria, smooth surface caries were evaluated and photographed under a stereomicroscope (Leica, Germany) [54]. Then, all caries lesions of the maxillary and mandible were exposed by a turbine cutter through the sagittal section and were photographed after a proper sonic bath. The caries lesions on sulcal and smooth surfaces were scored according to the Keyes method [54].

### 4.14. Statistical Analysis

One-way ANOVA and Tukey HSD tests were conducted to compare the differences between different treatment groups. The statistical analyses were mainly carried out using SPSS 20.0 (IBM, Chicago, IL, USA). The significance level (α) was set as 0.05.

## Figures and Tables

**Table 1:** The minimum inhibition concentration (MIC) and minimum bactericidal concentration (MBC) against *S. mutans*.

Tested Strain	Sulforaphene (SFE)		Chlorhexidine (CHX)	
	MIC ^1^ (mg/L)	MBC (mg/L)	MIC (mg/L)	MBC (mg/L)
*S. mutans* UA159	125.00 ± 0.00	>500	2.93 ± 1.07	15.63 ± 0.00
*S. mutans* GS-5	145.83 ± 51.03	>500	1.95 ± 0.00	11.72 ± 4.28
*S. mutans* COCC32-3	125.00 ± 0.00	>500	2.28 ± 0.80	11.72 ± 4.28
*S. mutans* COCC33-17	125.00 ± 0.00	>500	3.25 ± 2.37	15.63 ± 0.00
*S. mutans* COCC31-8	125.00 ± 0.00	>500	2.60 ± 1.01	15.63 ± 0.00
*S. mutans* COCC33-4	125.00 ± 0.00	>500	2.28 ± 0.80	15.63 ± 0.00
*S. mutans* COCC33-14	145.83 ± 51.03	>500	2.93 ± 1.07	15.63 ± 0.00
*S. mutans* COCC33-8	125.00 ± 0.00	>500	2.60 ± 1.01	15.63 ± 0.00

^1^ The data are shown as the means and standard deviations of six independent tests.

**Table 2:** Sequences of the primers used for reverse transcription and quantitative real-time PCR.

Gene	Forward (5′ → 3′)	Reverse (5′ → 3′)	Reference
16S rRNA	AGCGTTGTCCGGATTTATTG	CTACGCATTTCACCGCTACA	[48]
*ldh*	AAAAACCAGGCGAAACTCGC	CTGAACGCGCATCAACATCA	[49]
*eno*	GTTGAACTTCGCGATGGAGAT	GTCAAGTGCGATCATTGCTTTAT	[50]
*gtf*B	CACTATCGGCGGTTACGAAT	CAATTTGGAGCAAGTCAGCA	[48]
*gtf*C	GATGCTGCAAACTTCGAACA	TATTGACGCTGCGTTTCTTG	[48]

## Data Availability

The data that support the findings of this study are available from the corresponding author [Y.W.], upon reasonable request.
